# Profiling of mature-stage human breast milk cells identifies six unique lactocyte subpopulations

**DOI:** 10.1126/sciadv.abm6865

**Published:** 2022-06-29

**Authors:** John P. Gleeson, Namit Chaudhary, Katherine C. Fein, Rose Doerfler, Patricia Hredzak-Showalter, Kathryn A. Whitehead

**Affiliations:** 1 Department of Chemical Engineering, Carnegie Mellon University, Pittsburgh, PA 15213, USA.; 2 Department of Biomedical Engineering, Carnegie Mellon University, Pittsburgh, PA 15213, USA.

## Abstract

Breast milk is chock-full of nutrients, immunological factors, and cells that aid infant development. Maternal cells are the least studied breast milk component, and their unique properties are difficult to identify using traditional techniques. Here, we characterized the cells in mature-stage breast milk from healthy donors at the protein, gene, and transcriptome levels. Holistic analysis of flow cytometry, quantitative polymerase chain reaction, and single-cell RNA sequencing data identified the predominant cell population as epithelial with smaller populations of macrophages and T cells. Two percent of epithelial cells expressed four stem cell markers: SOX2, TRA-1-60, NANOG, and SSEA4. Furthermore, milk contained six distinct epithelial lactocyte subpopulations, including three previously unidentified subpopulations programmed toward mucosal defense and intestinal development. Pseudotime analysis delineated the differentiation pathways of epithelial progenitors. Together, these data define healthy human maternal breast milk cells and provide a basis for their application in maternal and infant medicine.

## INTRODUCTION

Although human infants are born immature (*1*, *2*), their diet of breast milk enables continued organ growth and development postpartum, particularly within the intestine (*3*). Human milk contains growth factors, immunomodulatory components, and bacterial and maternal cells. A substantial body of research has delineated the role of many nutritional components of milk, including proteins, fats, and sugars, with a primary goal of creating formula that recapitulates breast milk (*4*). There is also considerable interest in bacterial cells present in the maternal milk microbiome, which help to establish the infant gut microflora (*5*). By contrast, despite their abundance (~10^6^ cells/ml), the biology of breast milk cells and their potential role in infant development remain poorly understood.

The first step in determining the physiological relevance of breast milk cells is to identify and characterize them. Various breast milk cell types have been described to some degree in the literature, including lactocytes, immune cells, myoepithelial cells, and stem cells (*6*). Immune cells are the dominant population in early-stage milk (colostrum; <1 week after birth), whereas, in mature-stage milk from healthy mothers (>2 weeks postpartum), most of the cells are lactocytes, which are terminally differentiated milk-producing epithelial cells (*7*). Multipotent stem cells have also been identified in breast milk on the basis of the expression of several stem cell gene markers, including *p63* and *KLF4* (*8*, *9*). These cells were also differentiated into all three germ layers (*10*), a unique characteristic of pluripotent stem cells (*11*).

Although the roles of the maternal cells in breast milk are not yet clear, there is early evidence that they may participate in several vital functions. For example, bulk RNA sequencing (RNA-seq) studies showed that the maternal cells in colostrum express a high level of non-nutritive genes (iron binding, triglyceride catabolism, and protein digestion). Dominant gene expression then shifts in mature-stage milk to the two genes responsible for producing milk proteins—α-lactalbumin and β-casein (*12*). Establishing baseline concentrations and gene expression profiles for distinct breast milk cell types might also aid the development of diagnostics for the detection of cancer in the breastfeeding person (*13*).

In addition, there is a report that maternal stem and immune cells exit the gastrointestinal tract of the infant and functionally integrate into the infant’s organs, similarly to immune cells (*14*). These data open the possibility that maternal cells play a role in organ development and passive immunity. For example, necrotizing enterocolitis, an intestinal disease associated with the use of formula, affects ~7% of premature infants and leads to 1000 to 2000 infant deaths a year in the United States (*15*, *16*).

Given this mounting evidence that maternal breast milk cells are involved in infant development and may have properties amenable to cellular therapies and diagnostics (*17*), it is imperative that milk cells are clearly and comprehensively characterized. To that end, we were motivated to conduct a three-pronged analysis of milk cells with the broadest applicability to nursing people worldwide—fresh, mature-stage milk cells from healthy donors. The experiments reported here were designed to address the limitations of previous milk cell studies.

Specifically, numerous studies relied exclusively on flow cytometry to identify distinct milk cell populations (*10*). This is problematic because of variable fluorescent antibody quality and unavailability of antibodies for previously unidentified cell types in milk. Another limitation is that maternal breast milk cells are sometimes frozen after collection and before analysis (*13*). This can be problematic because some populations of breast milk cells do not survive the freezing process; thus, analysis biases against cell populations that are not sufficiently robust.

Through this work, we have circumvented these challenges by analyzing fresh maternal breast milk cells by flow cytometry, quantitative real-time polymerase chain reaction (RT-qPCR), and single-cell RNA-seq (scRNA-seq) simultaneously. Although there is one other study that characterized breast milk cells using scRNA-seq, it was performed on frozen breast milk samples from females with gestational diabetes (*13*). To our knowledge, our study is the first of its kind, as it holistically describes the complete population of maternal breast milk cells in mature-stage breast milk from healthy females. Because most lactating mothers are classified as healthy, these findings provide a broadly applicable reference dataset for maternal breast milk cells and lay the foundation for future studies on the role of these cells in infant development and their potential as therapeutic interventions.

## RESULTS

### Epithelial lactocytes are the most abundant cell population in breast milk

**Fig. 1.**
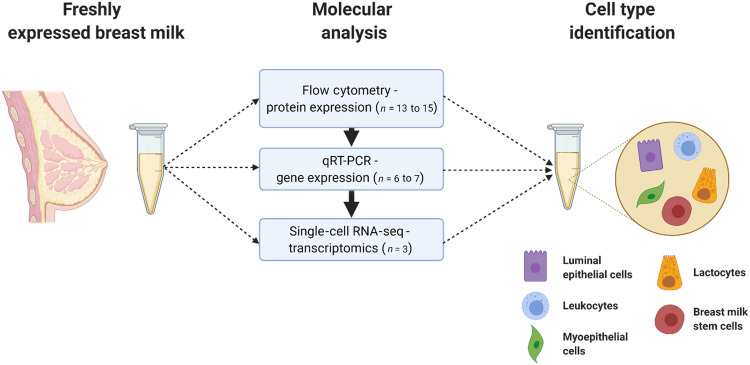
Maternal cells in breast milk were identified by protein, gene, and transcriptome analyses. Mature-stage breast milk from healthy donors was assessed for expression of cell marker proteins (e.g., EpCAM and CD45) in freshly isolated samples by flow cytometry. Then, RNA was isolated and used to conduct complementary gene expression analysis by RT-qPCR. Single-cell transcriptomics added depth to the analysis and defined the lactocyte population in breast milk.

For our initial analysis, we collected freshly expressed breast milk from donors to characterize the maternal cell populations using flow cytometry and RT-qPCR (Fig. 1). Although recruitment was open to all donors regardless of postpartum age, we received donations only of mature-stage milk (≥4 weeks, averaging 32 weeks postpartum; table S1) (*12*). We collected data on the donors’ health status (whether they or their infant were recently ill), their infants’ age, and gestational age at birth. Following the cell isolation process as previously reported (*10*), we assessed viability by flow cytometry using a fluorescent live-dead stain and observed a cell viability of 79.2 ± 6.9% (*n* = 10; fig. S1). The traditional trypan blue cell viability assay reported a high degree of false negatives due to the presence of milk fat globules; this assay should be avoided with breast milk cells. We prioritized sample analysis by flow cytometry, as that required smaller cell pellets to yield data, while RNA extraction required much higher amounts (table S1).

**Fig. 2.**
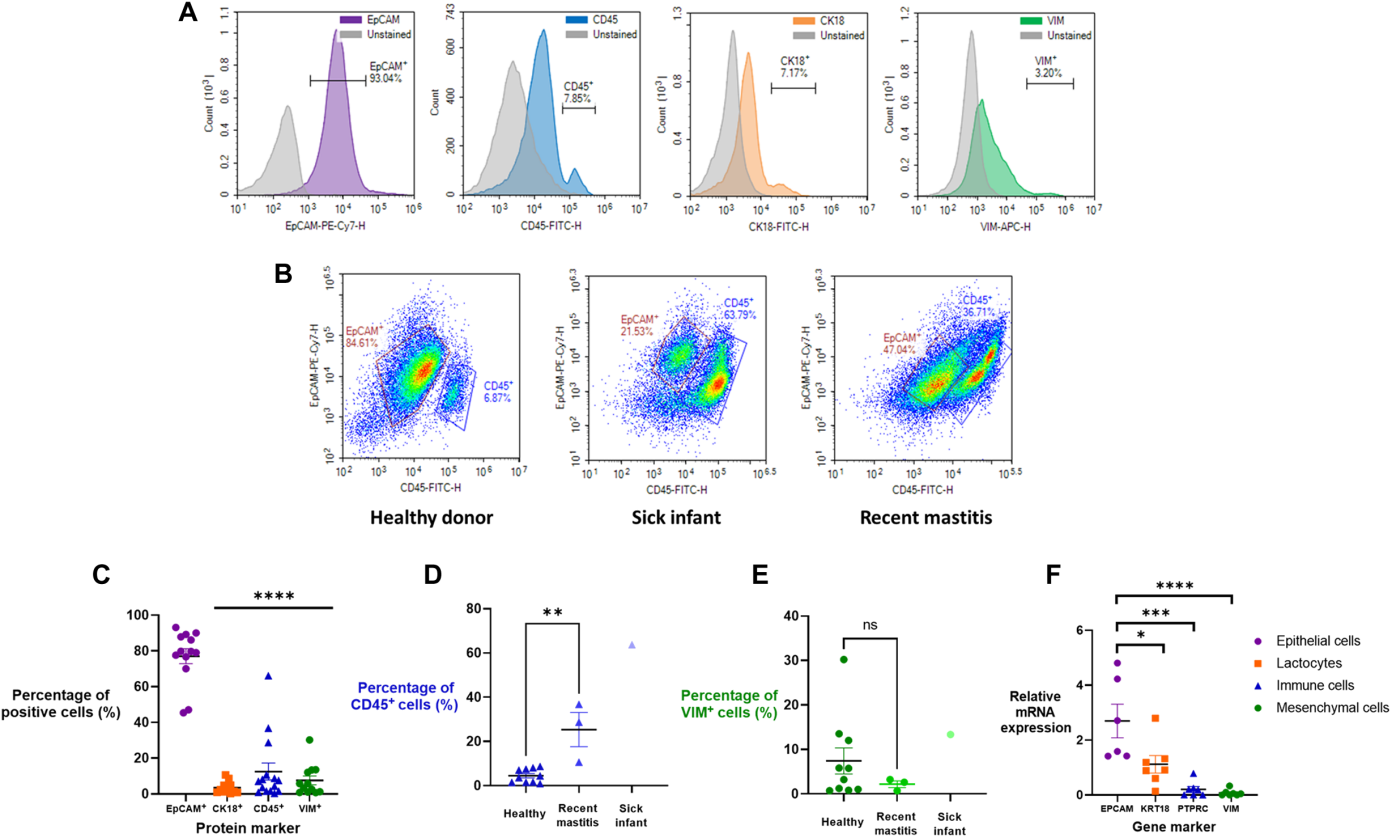
Mature-stage breast milk from donors contained epithelial lactocytes and a smaller population of immune cells. (**A**) Flow cytometry quantified expression of cell-specific markers: EpCAM^+^ (epithelial cells), CD45^+^ (immune cells), CK18^+^ (lactocytes), and VIM^+^ (mesenchymal cells). These data suggest that the CK18 antibody bounds poorly to lactocytes and likely underestimates true lactocyte percentages. FITC, fluorescein isothiocyanate. (**B**) The relative cellular composition in breast milk was affected by the health status of the mother and infant. Representative density plots are shown of EpCAM versus CD45 cell populations in milk from healthy donors (*n* = 10), donors with sick infants (*n* = 1), or donors with recent mastitis (*n* = 3). (**C**) Flow cytometry analysis of protein markers in all health statuses (*n* = 14) and the breakdown of (**D**) CD45^+^ immune cells and (**E**) VIM^+^ mesenchymal cells in different health statuses. (**F**) mRNA expression of gene markers corresponding to proteins in (C) indicates the presence of epithelial lactocytes (*n* = 6 to 7). Data are shown as means ± SEM. One-way analysis of variance (ANOVA) with Tukey’s multiple comparison test; *P < 0.05; **P < 0.01; ***P < 0.001; ****P < 0.0001. ns, not significant.

To understand the cell populations present, we selected surface markers for flow cytometry analysis based on previous studies: epithelial cells [epithelial cell adhesion molecule–positive (EpCAM^+^)], immune cells (CD45^+^), and mesenchymal cells [Vimentin (VIM^+^)] (*6*, *10*, *12*). We also assayed for lactocytes, which derive from epithelial cells, and thus are EpCAM^+^ in addition to being CK18^+^. The selected histograms indicate that the vast majority of breast milk cells are of epithelial origin (93%), with more modest populations of CK18^+^, CD45^+^, and VIM^+^ cells from healthy donors (*n* = 13) (Fig. 2A). Immune cell numbers increased from ~7% in milk from healthy donor-infant dyads to ~63 and ~36% when the infant (*n* = 1) or the mother (*n* = 3), respectively, had been sick (Fig. 2B). These flow cytometry data are shown for all donors in Fig. 2C and agree with previous reports, aside from the low percentage of cells expressing CK18 (*10*, *13*). In the case of illness, the number of donors was too low to run an appropriately powered statistical analysis but shows that, in the case of both recent mastitis and infant illness, there is an increase in immune cells (Fig. 2D). There was no statistically significant shift in the mesenchymal cell population regardless of health status (Fig. 2E). However, mRNA expression analysis shows reasonably high expression of *KRT18*, the gene associated with CK18 and lactocytes (Fig. 2F). This agrees with other reported profiles of lactocyte populations in breast milk regardless of the number of children a person has birthed (*13*, *18*). Accordingly, we concluded that the lactocyte population observed by flow cytometry was artificially low, likely because of poor binding affinity of the antibody. Gene expression of an immune cell gene, *PTPRC*, and the mesenchymal gene marker, *VIM*, were significantly lower in expression, consistent with the flow cytometry data.

### Stem-like transcription factors are highly expressed in 5% of breast milk cells

There has been a significant interest in breast milk as a potentially unique source of stem cells. Stem cells can be identified by the transcription factors SOX2, NANOG, and OCT4, which are considered the master regulators in stem cells, and by REX1, SSEA4, TRA-1-60, and KLF4, which are co-regulators of pluripotency (*19*). Hence, we asked whether the unknown fraction of epithelial cells in breast milk were stem like using flow cytometry and RT-qPCR.

**Fig. 3.**
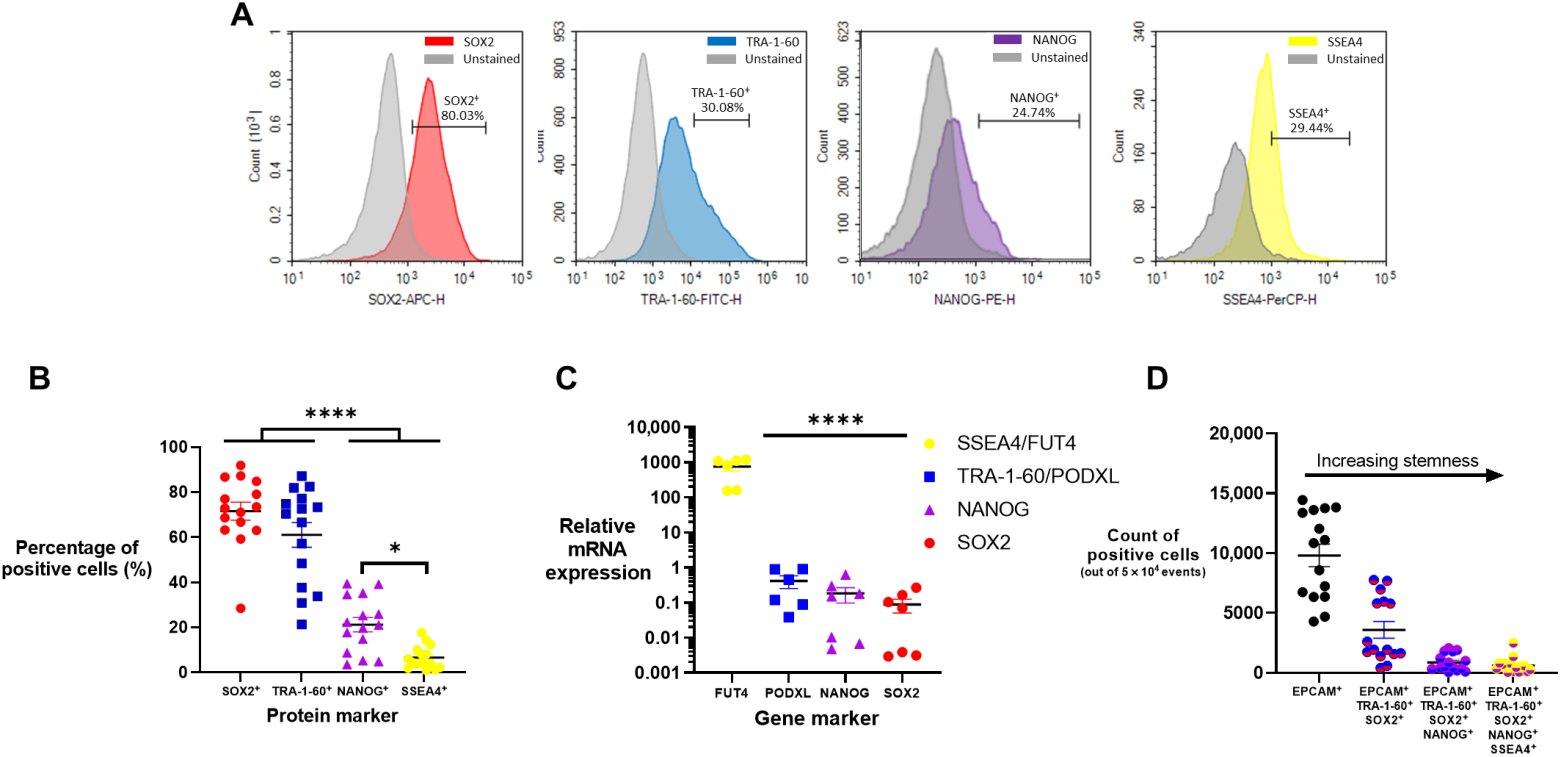
Stem-like transcription factors were expressed in 5% of breast milk cells. Four stem-like transcription factors were assessed in breast milk epithelial cells. (**A**) From flow cytometry analysis, representative histograms of SOX2^+^, TRA-1-60^+^, NANOG^+^, and SSEA4^+^ stained and unstained samples are shown. (**B**) Flow cytometry analysis of EpCAM^+^ cells (*n* = 15) indicated high expression of SOX2 and TRA-1-60 with moderate to low expression of NANOG and SSEA4, respectively. (**C**) mRNA expression relative to a housekeeping gene was quantified for the genes corresponding to the proteins in (B) (*n* = 7). (**D**) Flow cytometry analysis of EpCAM^+^ cells and their increasing stemness based on positive expression of multiple stem-like markers (*n* = 15). Data are means ± SEM. One-way ANOVA with Tukey’s multiple comparison test; *P < 0.05; ****P < 0.0001.

For flow cytometry, we gated on EpCAM^+^ cells and then measured the percentage of cells expressing SOX2, TRA-1-60, NANOG, and SSEA4 (Fig. 3A). Although we wanted to assess OCT4 as well, we were limited by the constraints of our flow panel. Ultimately, we deemed it more informative to have two master regulator markers and two pluripotency markers in our analysis. These experiments indicated that most epithelial cells were SOX2^+^ and TRA-1-60^+^ but significantly fewer were NANOG^+^ and SSEA1^+^ (Fig. 3, B and D).

We also conducted gene expression analysis by RT-qPCR for the genes encoding SOX2, TRA-1-60, NANOG, and SSEA4: *SOX2*, *PODXL*, *NANOG*, and *FUT4*, respectively. We quantified expression levels relative to the housekeeping gene, *EEF1A*, which was highly and uniformly expressed in all breast milk cells. Epithelial cells expressed 1000-fold greater *FUT4* mRNA than the other assessed genes, with decreasing expression of *PODXL* > *NANOG* > *SOX2* (Fig. 3C). The higher *FUT4* expression is likely due to its role in human milk oligosaccharide synthesis, so expression here may not be solely pluripotency related (*20*). To calculate the stem-like population, we used a gating strategy based on EpCAM^+^ cells (fig. S2) that subsequently gated on positivity for stem cell markers. To ensure accuracy in analysis, we verified the fluorescence minus one of each antibody (fig. S3) and the stem cell markers were verified in induced pluripotent stem cells (iPSCs) (fig. S4). This allowed us to calculate the percentage of cells that were positive for two or more stem cell markers (Fig. 3D). When considered together, these data indicate that ~5% of the epithelial cells were positive for all four transcription factors assessed.

### Single-cell transcriptomic analysis reveals unique lactocyte subtypes

Our flow cytometry and mRNA expression data suggest that breast milk cells are primarily epithelial with a moderate number of lactocytes and a small number of stem-like cells. However, only limited conclusions can be drawn from these two techniques, as the quality of flow data may have been influenced by antibody quality, and bulk gene expression data cannot delineate gene expression patterns in varied cell populations.

To overcome the latter shortcoming, we performed scRNA-seq to robustly quantify and classify the maternal cell types in milk. For these experiments, we identified six donors with similar cellular profiles by flow cytometry as determined by a first donation. These donors provided samples a second time, and we selected three of those donors (mean, 28 weeks postpartum) to advance to scRNA-seq experiments based on sample consistency and cell yield as determined by flow cytometry (table S1). Upon their third donation, we submitted samples from these three donors within 1 to 2 hours of expression for scRNA-seq analysis. In total, the transcriptomes of 26,922 maternal breast milk cells were sequenced, with a median of 1057 genes per cell.

**Fig. 4.**
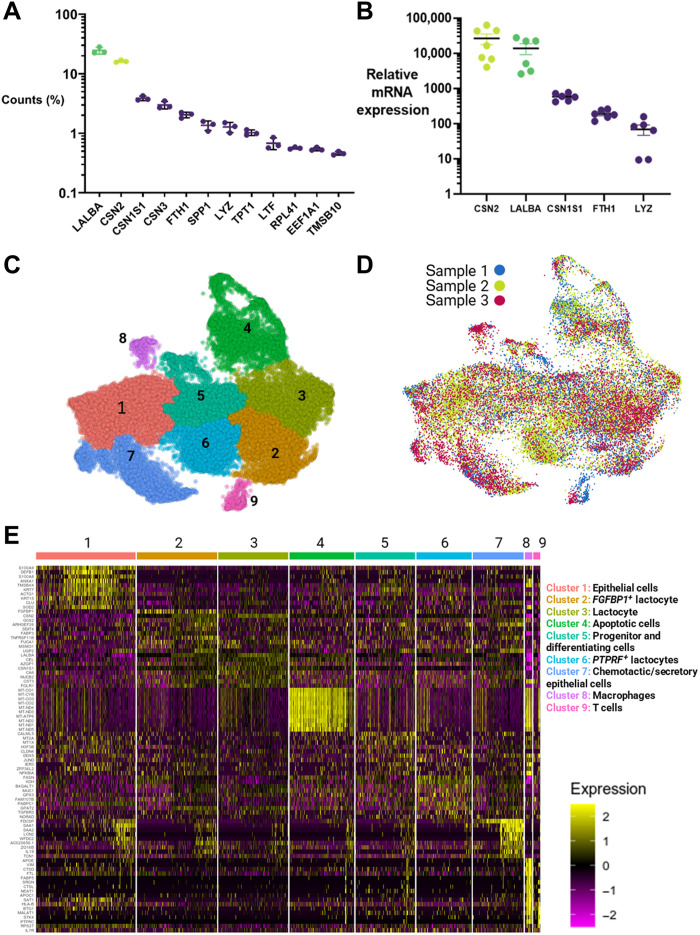
Single-cell transcriptome analysis identified breast milk cells as primarily epithelial-derived lactocytes, including three novel subpopulations involved in mucosal defense. (**A**) Fifty percent of the transcripts produced by mature breast milk cells from healthy donors corresponded to the milk-producing genes for lactalbumin (*LALBA*) and three types of caseins (*CSN2*, *CSN1S1*, and *CSN3*), *n* = 3. (**B**) RT-qPCR quantified mRNA expression relative to *EEF1A* expression for several top genes (*n* = 6). (**C**) scRNA-seq data generated a UMAP plot that identified one major population of cells (clusters 1 to 7) and two minor populations (clusters 8 and 9). (**D**) The UMAP cluster map is consistent across all donors (*n* = 3). Individual donor UMAP cluster maps are shown in fig. S5. (**E**) Each cluster had unique expression patterns of the top 10 marker genes. These patterns were used to identify specific cell types, including three previously unidentified types of epithelial lactocytes: *FGFBP1*^+^ lactocytes, *PTPRF*^+^ lactocytes, and chemotactic epithelial cells. Expression values are relative (unitless) and arbitrary. A higher-resolution image can be found in fig. S6.

Gene expression across all three donor samples was dominated by two genes, *LALBA* and *CSN2*, that accounted for >40% of gene counts (Fig. 4A). These two genes encode the proteins α-lactalbumin and β-casein, respectively, which are the two most abundant proteins in human milk (*21*). The genes identified by scRNA-seq were confirmed in a larger donor population by RT-qPCR (*n* = 7); high expression of *CSN2* and *LALBA* with decreasing expression of *CSN1S1* > *FTH1* > *LYZ*; and genes encoding the proteins αS1-casein, ferritin heavy chain 1, and lysozyme, respectively (Fig. 4B).

To identify the main cell types within the breast milk cell population that are generalizable across individuals, we performed a combined analysis of the scRNA-seq data using the Seurat and Monocle pipeline (*22*). This analysis identified one large group of cells comprising the contiguous clusters 1 to 7 and two smaller independent clusters (8 and 9) on the basis of uniform manifold approximation and projection (UMAP) clustering (Fig. 4C) (*23*). Comparison across donors showed that their cluster mappings were reasonably homogeneous (Fig. 4D), with notable variations in clusters 8 and 9 (fig. S7).

We then examined the differential expression of genes within each cluster (Fig. 4E), which identified two immune cell clusters, cluster 8 as macrophages (*CD68*^+^ and *ITGAX*^+^) and cluster 9 as T cells (*PTPRC*^+^ and *CD3E*^+^). Although the major cluster (1 to 7) was epithelial (*LALBA*^+^ and *EPCAM*^+^), there were unique subpopulations that we believe to be previously unidentified in human breast milk: *FGFBP1*^+^ lactocytes for intestinal development (cluster 2), fatty acid synthesizing and insulin-resistant *PTPRF*^+^ lactocytes (cluster 6), and secretory/chemotactic epithelial cells (cluster 7). Cluster 5 contained the cells that had undergone the least differentiation. These included a small population of progenitor-like cells, defined by high expression of *CD55* and *CLDN4* (*24*), and a larger population of differentiating cells, which are the precursors of epithelial cells and lactocytes. Last, cluster 4 comprised apoptotic epithelial cells, and it is unclear what cluster these cells belonged to before degradation.

**Fig. 5.**
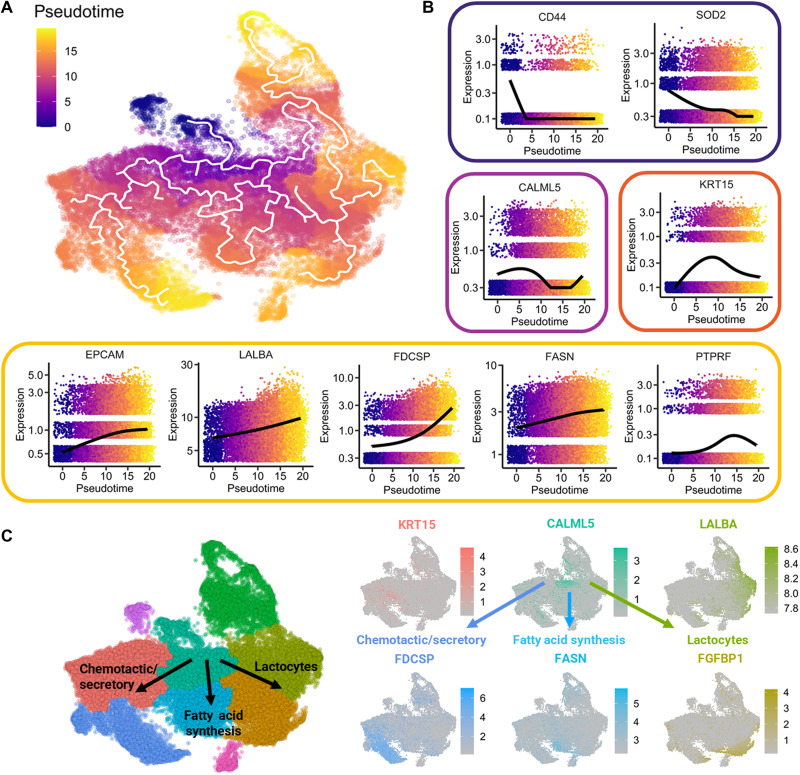
Pseudotime trajectory analysis identified the pathways by which breast milk epithelial cells differentiate into lactocytes. (**A**) Pseudotime trajectory analysis determined the pathway of differentiation of progenitor-like epithelial cells (starting node is located in the dark purple region). (**B**) As pseudotime progressed, the expression of progenitor genes subsided as cells differentiated into mature phenotypes. (**C**) On the basis of the pseudotime trajectories and gene expression kinetics, milk cells differentiated along three pathways into chemotactic/secretory epithelial cells, fatty acid–synthesizing cells, and mature lactocytes. Each pathway was associated with the overexpression of key genes compared to the other cell types, including *KRT15* (cluster 1), *FDCSP* (cluster 7), *FASN* (cluster 6), *LALBA* (cluster 2), and *FGFBP1* (cluster 3).

To determine the pathway of differentiation of progenitor-like epithelial cells, we performed pseudotime trajectory analysis (Fig. 5). Figure 5A shows the differentiation pathways of the breast milk cells, originating from the most progenitor-like cells, which are part of cluster 5. The starting node is found in the top, deep purple portion of cluster 5. As pseudotime progresses, the cells differentiate along several distinct pathways, with partially and terminally differentiated cells appearing in reddish orange and yellow, respectively. Figure 5B shows the spatiotemporal gene expression profiles of nine selected cell markers. High expression of genes associated with progenitor cells or stemness (e.g., *CD44* and *SOD2*) occurred earliest in pseudotime and corresponded with cells in cluster 5 (*25*–*27*), with *SOD2* peaking in cluster 1. These progenitor cells briefly expressed *CALML5*, which was exclusively expressed by differentiating epithelial cells, before they diverged into several cell types. Differentiated cells, which appeared in late pseudotime, included mature lactocytes in clusters 2 and 3, lipid and fatty acid synthesizing cells in cluster 6, and chemotactic/secretory epithelial cells in clusters 1 and 7 (Fig. 5C).

Last, we examined the expression homogeneity of selected markers across seven donors (fig. S7), two of which were included in our scRNA-seq (table S1). *SOD2* and *CALML5* mRNAs were moderately and homogeneously expressed (fig. S7, A and B), suggesting that progenitor and epithelial cell populations are consistent across donors. By contrast, the chemotactic epithelial marker *FDCSP* had 30- and 100-fold higher expression in two donors (fig. S7D). These data suggest that lactocytes are homogeneous across donors, while the specialized subtypes may be enriched for a specific need of the feeding infant, such as intestinal stem cell development (*FGFBP1*^+^ lactocytes; cluster 2), insulin resistance (*PTPRF*^+^ lactocytes; cluster 6), or intestinal immune defense (chemotactic epithelial cells; cluster 7). It should be noted that one of the pseudotime branches terminates in the apoptotic cell cluster 4. This affects the pseudotime expression of genes that are up-regulated in the apoptotic cluster, such as *LALBA*, *FASN*, and *CALML5*. This impact is not apparent in the pseudotime expression of *LALBA* and *FASN* because of their overall high expression levels. *CALML5* peaks in the undifferentiated progenitor-like cells and decreases over time but has a smaller spike in the apoptotic cluster.

## DISCUSSION

Through this work, we have provided a detailed characterization of the cellular population in healthy mature-stage breast milk. We confirmed that most cells were epithelial lactocytes and that ~5% of breast milk cells are stem like. Single-cell transcriptomic analysis identified previously unidentified subpopulations of lactocytes programmed toward mucosal defense and intestinal development; however, this requires further in vitro assessment of efficacy.

Most research on human breast milk cells has focused on the characterization of stem-like cells present in the milk (*14*, *17*, *28*, *29*). The most thorough of these studies isolated stem-like cells expressing multiple stem transcription factors (OCT4^+^, SOX2^+^, and NANOG^+^) and differentiated them into all three germ layers (*10*). Unfortunately, this previous study was unable to determine the percentage of cells positive for more than one marker, because the antibodies used in flow cytometry experiments were all fluorescein isothiocyanate labeled. To our knowledge, we are the first to report that the percentage population of stem-like cells in breast milk is ~5% (SOX2^+^/TRA-1-60^+^/NANOG^+^/SSEA4^+^) (Fig. 3).

Single-cell transcriptomic analysis did not identify a cluster of stem-like cells within our breast milk cell population, nor were we able to isolate any clusters with high expression of the typical stem-like transcription factors, which is in agreement with recent publications (*30*). These data are not necessarily contradictory to our flow cytometry and qPCR data. One limitation of scRNA-seq is that information about rare genes can be obscured because of several factors, including GC content, secondary RNA structure, RNA sheering process, and RNA-seq library prep. For example, Rachinger and colleagues (*31*) recently demonstrated this issue when analyzing the SOX family of genes. While complementary DNA (cDNA) microarray, RT-PCR, and Western blot data concurred, RNA-seq data did not. In addition, Zhang *et al*. (*32*) have stated that genes identified by RNA-seq must be validated by qPCR, while the opposite is not necessary. With these technique shortcomings in mind along with previously published work (*10*), we therefore conclude that milk contains a stem-like cell population. Future analysis of this population may benefit from combination transcriptomic and proteomic approaches (*33*) or mass cytometry [Cytometry by time of flight (CyTOF)] in combination with our reference dataset (*34*).

Our findings are generally aligned with those reported from the scRNA-seq analysis of two donors with gestational diabetes (*13*) and a longitudinal scRNA-seq lactation study (*30*). Differences observed might be due to the number of cells analyzed, number of donors, and/or our use of fresh rather than frozen samples. The use of fresh cells assures that results are not biased toward more robust milk cells that survive freeze/thaw cycles. Furthermore, freeze/thaw processes have been linked to altered gene expression and translation in human primary cells (*35*, *36*). For example, Lee *et al*. (*37*) demonstrated that fresh peripheral blood mononuclear cells had a higher unique molecular identifier, higher medium genes per cell, and altered scRNA-seq cell clustering compared to cells that were freeze-thawed. Nyquist and colleagues (*30*) found that freezing breast milk cell samples led to a decrease in quality data and recommend processing fresh or storage at 4°C overnight.

These methodological differences enabled our identification of previously unidentified subpopulations: *FGFBP1*^+^ lactocytes (cluster 2), *PTPRF*^+^ lactocytes (cluster 6), and chemotactic epithelial cells (cluster 7; Fig. 4E). Regarding cluster 2, *FGFBP1* is a soluble carrier protein that binds to fibroblast growth factor (FGF), which plays a key role in intestinal development and disease regulation (*38*, *39*). Cluster 2 also expressed *TNFRSF11B*, which activates the Wnt/β-catenin pathway (essential for intestinal stem cell proliferation) (*40*) and regulates the differentiation of immune sampling M cells (*41*).

The *PTPRF*^+^ lactocytes highly expressed *MUC1*, *TGFBR3*, and *PTPRF*. *PTPRF* overexpression in breast milk cells might be linked to insulin-resistant mothers (*12*). *MUC1* secreted in breast milk binds to the lectin domain of dendritic cells found in infant intestines, preventing pathogenic interactions (*42*). *TGFBR3* plays a key role in activation and inhibition of T and B cells, respectively (*43*). In summary, this population may contribute to maternal insulin resistance signaling and intestinal mucosal defense in the infant.

Last, chemotactic epithelial cells (cluster 7) had high expression of *FDCSP*, *IL19*, *SAA1*, *WFDC2*, *TCN1*, and *LCN2*. *FDCSP* is considered an ancestral precursor gene to *CSN3*, which produces the milk protein κ-casein (*44*, *45*). The other genes with high expression in this cluster appear to confer mucosal defense, chemokine signaling, and intestinal immune maturation. For example, interleukin-19 expression was increased in the milk cells of lactating females with mastitis that had been treated with oral probiotics (*46*). The bioactive protein product of *SAA1* in human milk confers protection to the neonate in part by intestinal host defense and Th17 T-cells activation (*47*). In addition, *WFDC2* contributes to innate immune defenses of the lung, nasal, and oral cavities (*48*, *49*), and *LCN2* expression occurs in response to small bowel villous disruption (*50*). Assessing the functionality of these cells was beyond the scope of this study, given the need for custom antibodies to facilitate cell sorting. Once isolated, it would be possible to determine the response of these cells to external stimuli (cytokines, immune cells, etc.) and their interaction to oral mucosa and intestinal epithelium (*51*, *52*). In our study, collection of donor data was blinded to anything beyond health status at the time of milk donation, infant age, and gestational age at birth. Age, ethnicity, and diet can contribute to the nutritional aspects of breast milk, and future studies should delve into the impact of these factors on the breast milk cell population (*53*, *54*).

Bach and colleagues (*55*) suggested that mammary epithelial cells should be conceptualized as being part of a continuous spectrum of differentiation, and our results support this. Overall, our data suggest that the unique lactocyte subtypes identified here by scRNA-seq are temporally and uniquely suited to the health and development of mother-infant dyads and strengthen the infants’ immune system during early development. In addition, we showed (with limited statistical significance; Fig. 2, C and D) that the immune cell population in breast milk is modulated on the basis of both the mother and infant’s health status. Hassiotou *et al*. (*7*) reported that up to 94% of the total breast milk cell population consists of leukocytes in the case of infection, and Trend *et al*. (*56*) reported a decrease in basophils in mature breast milk in people with infections. This immune cell population in breast milk could be exploited for therapeutic applications such as chimeric antigen receptor (CAR) T cell therapy (*57*).

These findings have provided a genetic fingerprint for the cells in healthy, mature-stage breast milk and a framework for examining the functionality of these cell types in biologic, prophylactic, and therapeutic contexts. Future work will determine the role of the subpopulations identified here in healthy and sick infants and apply those learnings to the development of novel, oral therapeutic interventions for gastrointestinal and other infantile diseases.

## MATERIALS AND METHODS

### Breast milk sample collection and cell isolation

This study was approved by Carnegie Mellon University Institutional Review Board under the protocol number STUDY2019_00000084. iPSCs used for stem cell flow cytometry marker validation were obtained from Cedars-Sinai Medical Center’s iPSC Core (cell line number CS688iCTR). Participants were recruited via flyers and social media advertisements in mother and infant groups in the greater Pittsburgh area. All participants provided informed written consent and provided data on health status at the time of milk donation, infant age, and gestational age at birth. Pump-expressed mature breast milk was obtained from each participant at lactation suites at Carnegie Mellon University, placed on ice, and transported to the laboratory immediately after expression for analysis. Breast milk samples were processed as previously reported (*10*). In brief, breast milk samples were diluted 1:1 with sterile phosphate-buffered saline (PBS; Life Technologies), centrifuged, washed, and resuspended in PBS before centrifugation three times. The resultant breast milk cell pellet was then processed for scRNA-seq, RT-qPCR, or flow cytometry.

### Cell viability and flow cytometry analysis

Cell pellets were resuspended in 100 μl of 1% fetal bovine serum (FBS; VWR) and incubated with either trypan blue (Invitrogen) or LIVE/DEAD Fixable Yellow stain (Invitrogen) for 5 min at room temperature and 30 min at 37°C, respectively. Fixable Yellow samples were subsequently fixed using Flow Cytometry Fixation Buffer (R&D Systems), centrifuged, and resuspended in 1% FBS in PBS. Trypan blue cell viability was measured using Countess II Automated Cell Counter (Thermo Fisher Scientific). For flow cytometry, samples were fixed in fixation buffer for 30 min at 4°C, washed with permeabilization buffer (R&D Systems), centrifuged, and incubated with fluorescent antibodies (table S2) in permeabilization buffer at 4°C for 1 hour. Samples were then washed with 10% FBS in PBS, centrifuged, and resuspended in 10% FBS in PBS. Appropriate negative internal controls were used. Data acquisition was done with a NovoCyte 3000 (ACEA Biosciences) and data analysis with NovoExpress.

### Gene expression analysis by RT-qPCR

Cell pellets were lysed in buffer RLT and mixed with an equal volume of 100% ethanol, and the RNA was extracted with an RNeasy mini kit (QIAGEN). cDNA was generated from 1500 ng of RNA using High-Capacity cDNA Reverse Transcription Kit (Applied Biosystems). RT-qPCR was performed using SYBR Select Master Mix (Applied Biosystems) on a ViiA 7 Real-Time PCR System (Applied Biosystems). Primer sequences are presented in table S3.

### Single-cell RNA-seq

Cells were processed with Chromium Next GEM Single Cell 3′ GEM Library and Gel Bead Kit version 3.1 (10× Genomics) according to the manufacturer’s instructions. In brief, cells were diluted in master mix containing reverse transcription reagents and primer and were transferred to the Chromium chip with gel beads and partitioning oil for preparation of nanoliter-scale Gel Bead-In-Emulsions (GEMs). Reverse transcription produced cDNA with a cellular 10× barcode and unique molecular identifier (UMI), and the cDNA was recovered with Dynabeads MyOne SILANE magnetic beads. The cDNA was then subjected to 8 to 14 cycles of amplification before cleanup using SPRIselect Reagent (Beckman Coulter). Total cDNA yield was calculated following quality assessment (High Sensitivity D5000 ScreenTape, Agilent) and fluorometric quantitation (Qubit, Thermo Fisher Scientific). Libraries were constructed by enzymatic fragmentation, end repair, and A-tailing according to the manufacturer’s instructions. Two rounds of cleanup were performed: First, a double-sided SPRIselect Reagent (Beckman Coulter) prepared samples for adapter ligation. Then, another round of magnetic bead cleanup was performed before sample indexing PCR for 5 to 16 cycles depending on the cDNA input for library construction. A further double-sided size selection produced libraries of 400 to 600 base pairs (bp), which were quantified for paired-end sequencing (26 bp × 98 bp). The 10× single-cell RNA libraries quality was checked using Fragment Analyzer (NGS Fragment Kit, Agilent), and they were quantified by qPCR (KAPA qPCR quantification kit, Kapa Biosystems). The libraries were then normalized, pooled, and sequenced on a NovaSeq 6000 platform (Illumina) with S1 100-cycles kit (read 1: 28 bp and read 2: 91 bp).

### scRNA-seq analysis

All reads were processed using the 10× cellranger pipeline. In brief, reads were demultiplexed, using the cellranger mkfastq function. Demultiplexed reads from all lanes were aligned to the GRCh38 genome, followed by filtering, barcode counting, and UMI counting using the cellranger count function. After constructing the gene expression matrix, cells were processed using Seurat and Monocle packages. Cells were filtered to remove low-quality cells (<400 genes), doublets (>5000 genes), and cells with high mitochondrial fraction (>25%). Cells were assigned cell cycle scores based on the presence of S, G_1_, and G_2_-M genes. The filtered subset was normalized and scaled using Seurat’s SCT pipeline. Mitochondrial percentage, cell cycle scores, gene counts, and UMI counts were regressed out. Samples from all three donors were integrated using Seurat’s SCTransform integration pipeline. Highly variable genes from all 26,922 cells were used as features for principal components analysis (PCA) using the RunPCA function. The first 50 principal components were used for UMAP dimensionality reduction using the RunUMAP function. The integrated RNA counts from the Seurat object along with the first 50 principal components and the UMAP coordinates were transferred to a cell_data_set for clustering and trajectory inference using monocle3. Cells were clustered using the Leiden algorithm in monocle using the cluster_cells function. For trajectory inference, the principal graph was learned using the learn_graph function. Cells were ordered in pseudotime by choosing the root node using the order_cells function. A node in the epithelial cell cluster with high expression of Keratin 7 (KRT7) and Keratin 18 (KRT18) was chosen as the root node. Gene dynamics as a function of pseudotime were analyzed using the plot_genes_in_pseudotime function. Last, the clusters from monocle3 were added to the Seurat object’s metadata. Differentially expressed genes were identified using the Model-based Analysis of Single-cell Transcriptomics (MAST) algorithm with the FindAllMarkers function. Gene expression levels in Fig. 4E and fig. S4 are calculated relative to one another and are unitless.

### Statistical analysis

All data are represented as means ± SEM. Statistical analysis was carried out using Prism 8 software (GraphPad) using Student’s *t* test and one-way analysis of variance (ANOVA) with Bonferroni’s post hoc tests. A significant difference was defined as *P* < 0.05.
