# Supplementary material for: Profiling of mature-stage human breast milk cells identifies six unique lactocyte subpopulations
Source: Sci Adv. 2022 Jun 29;8(26):eabm6865. doi: 10.1126/sciadv.abm6865 (PMC9242445; doi:10.1126/sciadv.abm6865)
Supplement: Supplementary file 1 — Figs. S1 to S7 Tables S1 to S3 [file sciadv.abm6865_sm.pdf]

Science Advances
AAAS

Supplementary Materials for

Profiling of mature-stage human breast milk cells identifies six unique lactocyte subpopulations

John P. Gleeson *et al.*

Corresponding author: Kathryn A. Whitehead, kawhite@cmu.edu

Sci. Adv. **8**, eabm6865 (2022)
DOI: 10.1126/sciadv.abm6865

This PDF file includes:

Figs. S1 to S7
Tables S1 to S3

Fig. S1 – Cells isolated from the breastmilk of 12 unique donors were 40 to 95% viable with an average viability of 70%. Viability was measured using flow cytometry LIVE/DEAD™ Fixable Yellow stain (79.2% ± 6.9%; N = 10).

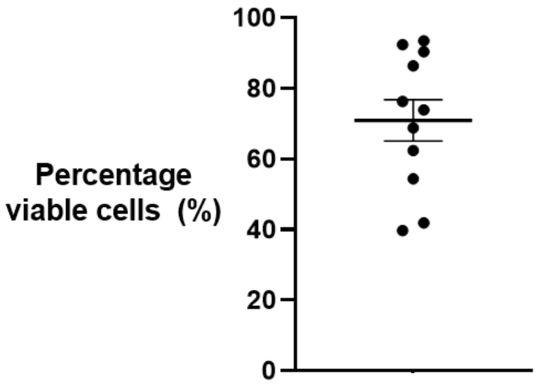

Fig. S2 – Representative gating strategy for multicolor flow cytometry analysis. Top panel shows the gates selected for unstained samples, the middle panel shows the gating in the presence of multicolor antibodies. The gating strategy selects for singlets and then selects for EpCAM+ cells. Of these, they are gated for double positive initially of SOX2+ and TRA-1-60+, and these double positive gated cells are subsequently gated for double positive NANOG+ and SSEA4+.

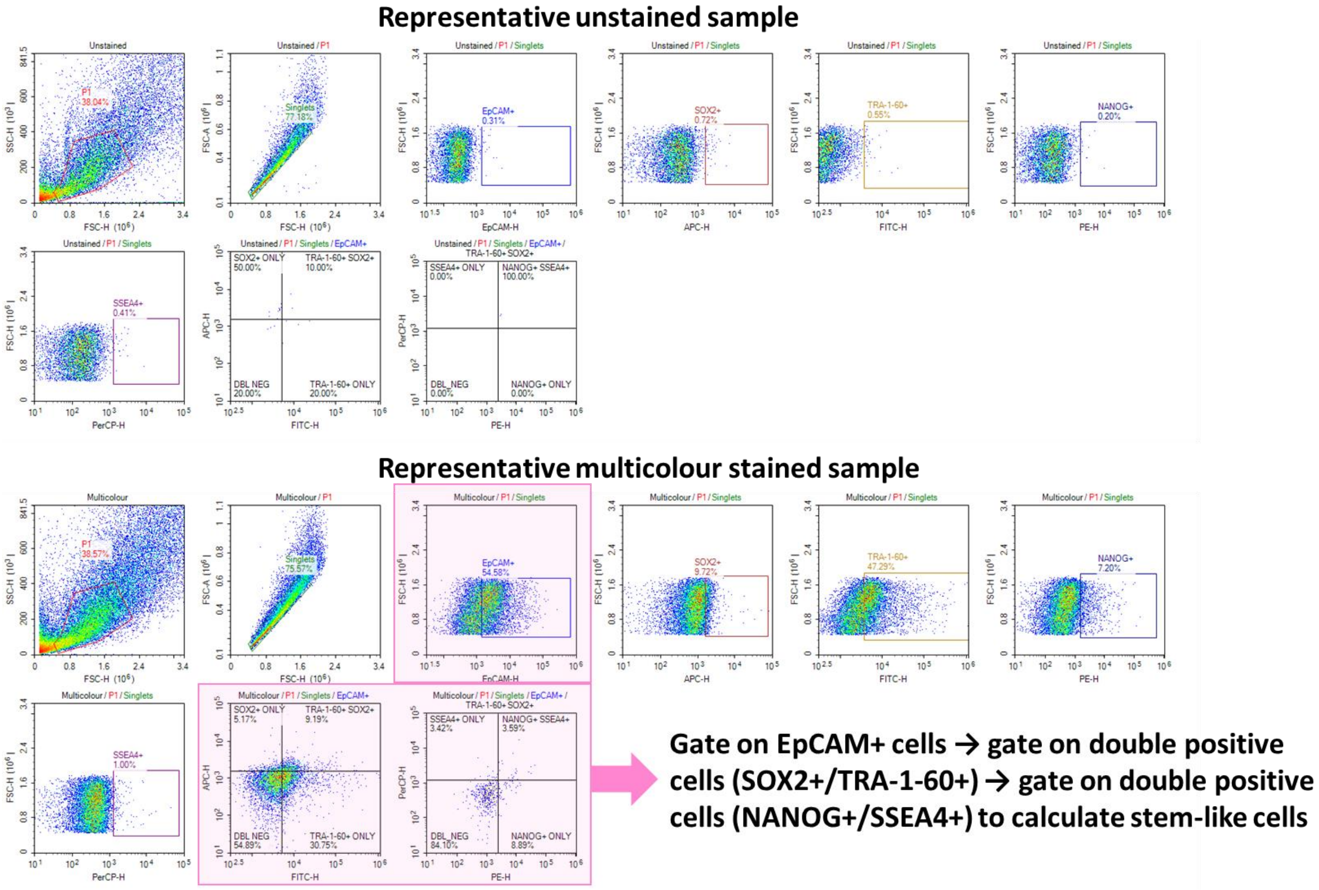

Fig. S3 – Representative fluorescence minus one (FMO) multicolor flow cytometry analysis on human breastmilk cells (n=2). Each sample contained either all of the antibodies in the panel (multicolor), removal of the antibody of interest (FMO), or no fluorescent antibodies (unstained).

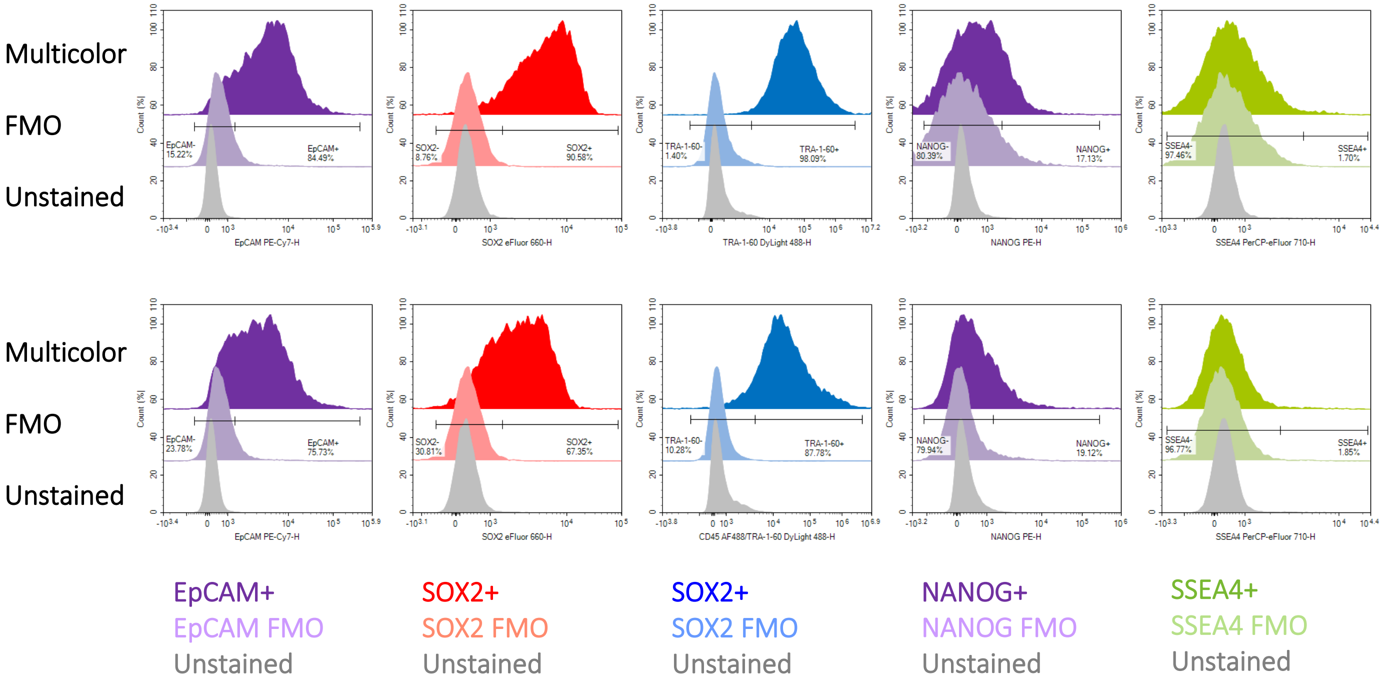

Fig. S4 – Representative validation of stem cell fluorescent flow cytometry markers in induced pluripotent stem cells (iPSC)

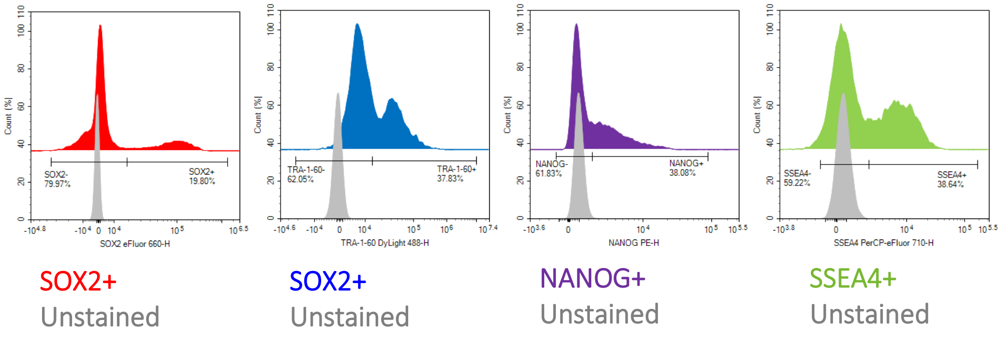

Fig. S5 – Single cell RNAseq data generated a UMAP plot in Fig. 4C/D. The UMAP cluster is consistent across donors with some variations shown below in individual donor UMAP plots.

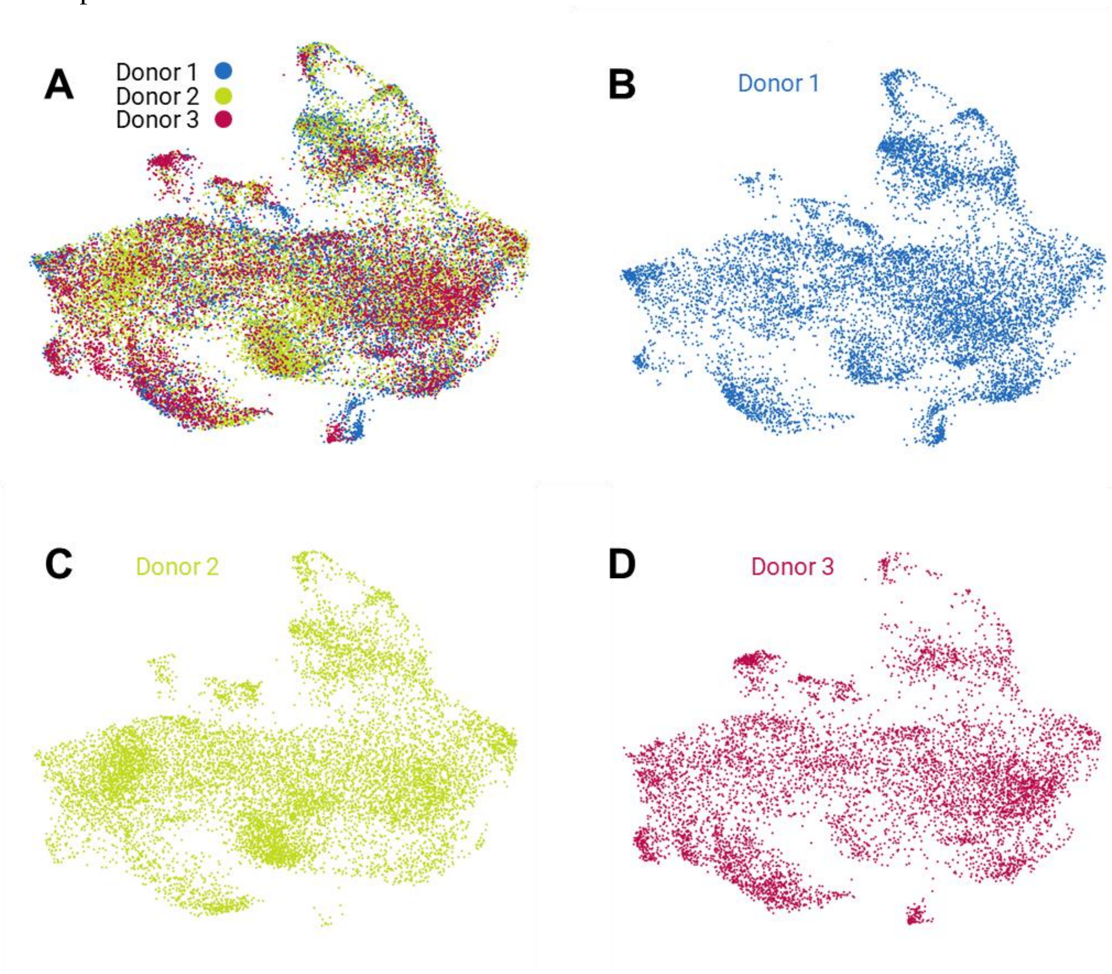

Fig. S6 – Higher quality cluster heatmap shown in Fig. 4E.

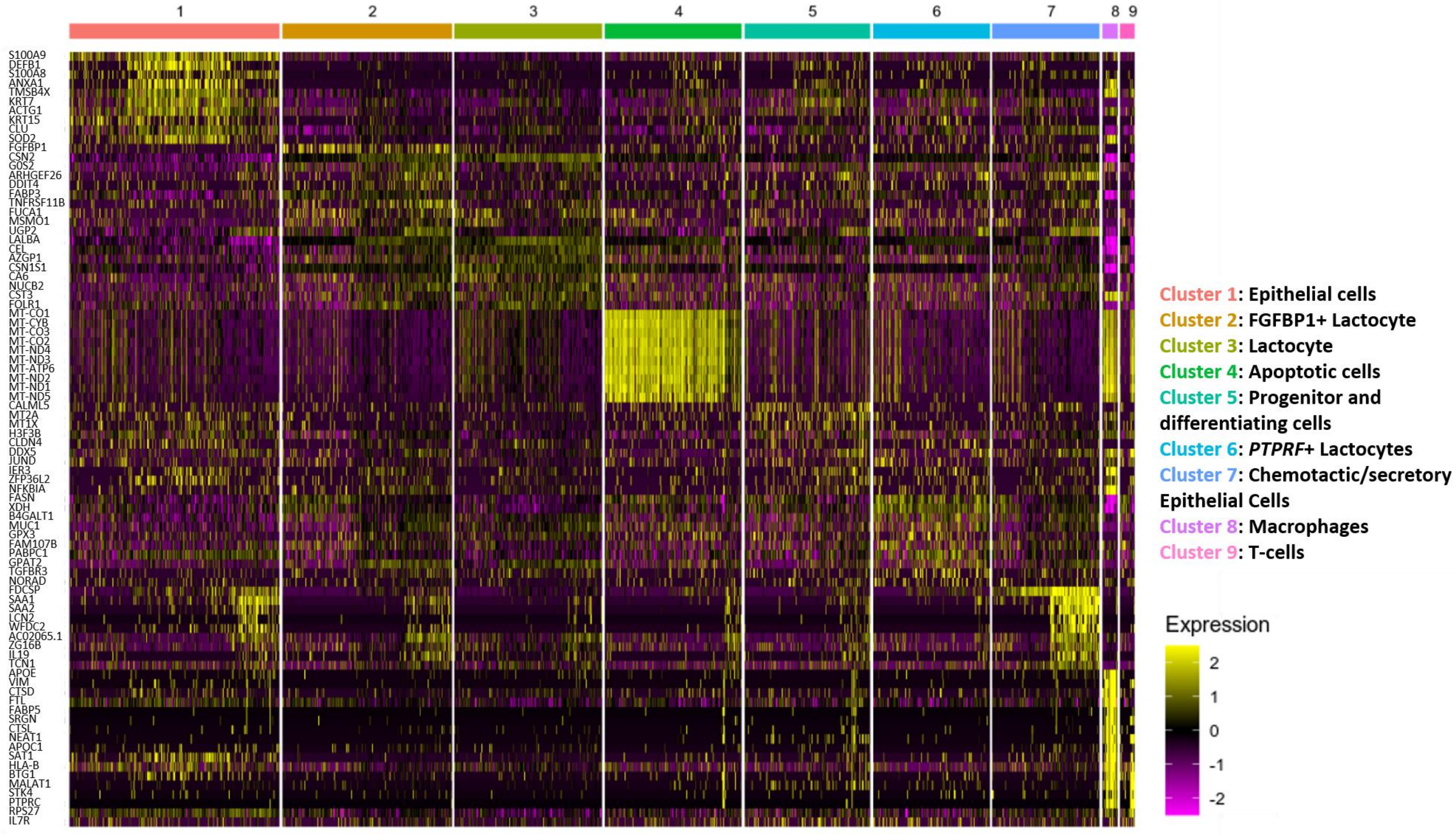
1
2
3
4
5
6
7
8
9
S100A9
DEFB1
S100A8
ANXA1
TMSB4X
KRT7
ACTG1
KRT15
CLU
SOD2
FGFBP1
CSN2
G0S2
ARHGEF26
DDIT4
FABP3
TNFRSF11B
FUCA1
MSMO1
UGP2
LALBA
CEL
AZGP1
CSN1S1
CA6
NUCB2
CST3
FOLR1
MT-CO1
MT-CYB
MT-CO3
MT-CO2
MT-ND4
MT-ND3
MT-ATP6
MT-ND2
MT-ND1
MT-ND5
CALML5
MT2A
MT1X
H3F3B
CLDN4
DDX5
JUND
IER3
ZFP36L2
NFKBIA
FASN
XDH
B4GALT1
MUC1
GPX3
FAM107B
PABPC1
GPAT2
TGFBR3
NORAD
FDCSP
SAA1
SAA2
LCN2
WFDC2
AC02065.1
ZG16B
IL19
TCN1
APOE
VIM
CTSD
FTL
FABP5
SRGN
CTSL
NEAT1
APOC1
SAT1
HLA-B
BTG1
MALAT1
STK4
PTPRC
RPS27
IL7R
Cluster 1: Epithelial cells
Cluster 2: FGFBP1+ Lactocyte
Cluster 3: Lactocyte
Cluster 4: Apoptotic cells
Cluster 5: Progenitor and differentiating cells
Cluster 6: *PTPRF*+ Lactocytes
Cluster 7: Chemotactic/secretory Epithelial Cells
Cluster 8: Macrophages
Cluster 9: T-cells
Expression
2
1
0
-1
-2

Fig. S7 – Cluster marker mRNA expression in a larger donor pool is relatively consistent (A) UMAP localization and corresponding mRNA expression of epithelial cell marker, progenitor cells (*SOD2*; cluster 1/5) and differentiating cell marker (*CALML5*: cluster 5) are consistently expressed across multiple donor samples. Markers for specialized sub-types are enriched in specific donors (C; *PTPRF*+ lactocytes) and (D; chemotactic epithelial cells). UMAP analysis (N = 3) and RT-qPCR (N = 7).

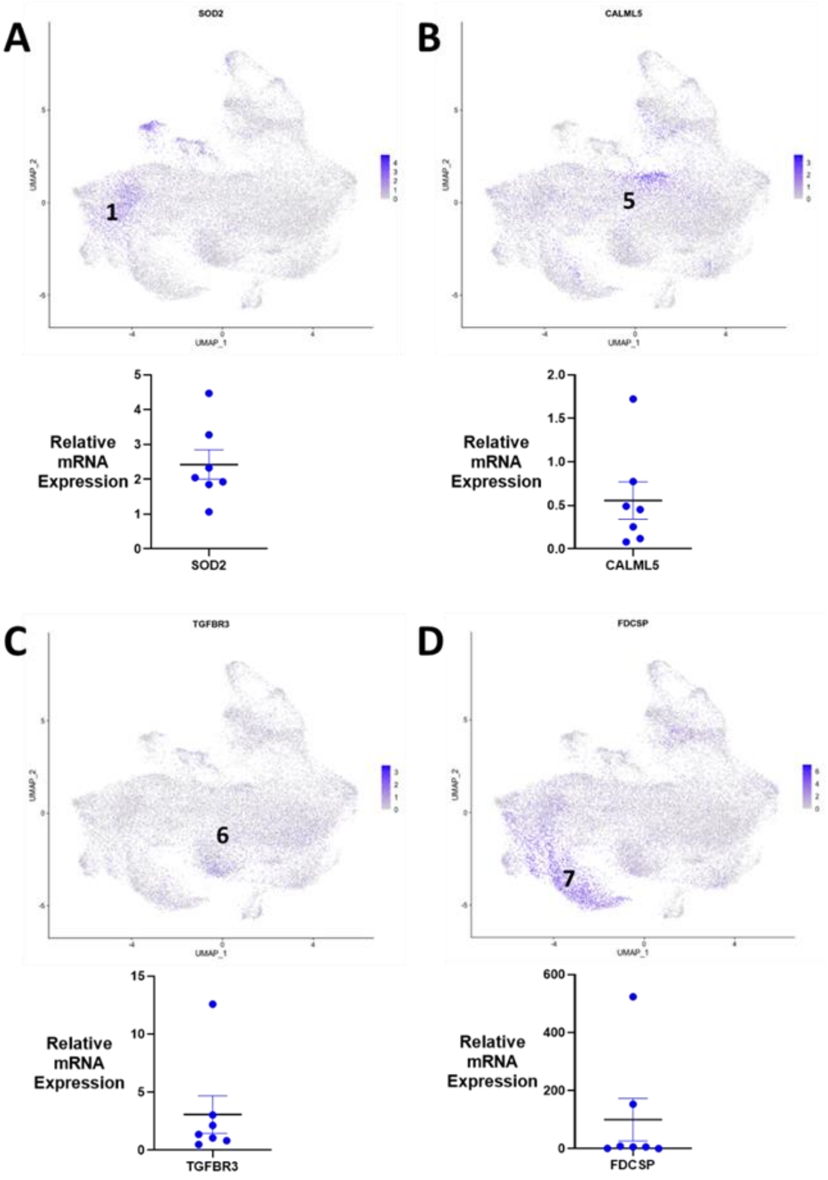

Table S1. Donor characteristics and experimental groups

Donor ID	Weeks Postpartum	Health status	Viability assay	Cell type flow cytometry	Cell type qPCR	Stem cell flow cytometry	Stem cell qPCR	scRNAseq	RNAseq qPCR
2.1	18	H		Y	Y	Y	Y		Y
2.2	28	H		Y		Y			
2.3	31	H	Y					Y	
4	38	H		Y		Y			
5	83	H	Y	Y		Y			
6.1	12	H		Y	Y	Y	Y		Y
6.2	19	H		Y		Y			
6.3	20	H	Y					Y	
8.1	39	H	Y	Y		Y			
8.2	44	H		Y		Y			Y
9	21	M	Y	Y		Y			
11	11	M	Y	Y	Y	Y	Y		Y
12.1	29	H		Y		Y			
12.2	32	H		Y		Y			
12.3	33	H	Y					Y	
13	92	H	Y	Y	Y	Y	Y		Y
17	6	S	Y	Y					
18	5	H		Y	Y	Y	Y		Y
19	36	M		Y					
21	40	H	Y	Y	Y	Y	Y		Y

Health status: H = healthy mother and infant, M = recent mastitis infection, and S = infant currently sick.

Table S2. Flow cytometry antibodies used in analysis

**Marker**	**Fluorophore**	**Company**	**Cat. No**
EpCAM	PE-Cy7	eBioscience	25-9326-42
CD45	Alexa Fluor® 488	R&D Systems	FAB1430G
Vimentin	APC	Invitrogen	MA528601
CK18	FITC	Invitrogen	MA110327
aSMA	Alexa Fluor® 594	R&D Systems	IC1420T-100
SOX2	eFluor® 660	eBioscience	50-9811-82
TRA-1-60	DyLight® 488	Invitrogen	MA1023D488X
Nanog	PE	Invitrogen	PA546891
SSEA4	PerCP-eFluor® 710	eBioscience	46-8843-42
CD49f	eFluor® 450	eBioscience	48-0495-82

Table S3. Primer sequences for RT-qPCR

Gene	Forward	Reverse
EEF1A	ATCTCAGGCTGACTGTGCTG	AGTGTGTAAGCCAGAAGGGC
EPCAM	GCTGGCCGTAAACTGCTTTG	ACATTTGGCAGCCAGCTTTG
PTPRC	TGAAATGTGTATGCACCTATTGAAA	TGGGGCCTGTAAAAGTGTCC
VIM	CGCACATTCGAGCAAAGACA	ACAAGAGCGCCCCTAAGTTT
KRT18	AAAGCCTGAGTCCTGTCCTTTC	GGTGAAGCTCATGCTGTCCG
FUT4	GGGGGTTCTTCCTCACCTTG	ATATGGCCTGTGGCAGATGG
PODXL	TCAGACCGTGGTCGTCAAAG	TTCATGTCACTGACCCCTGC
SOX2	TACAGCATGATGCAGGACCA	CCGTTCATGTAGGTCTGCGA
NANOG	ACATGAGTACTGCTTTAGTTGGT	TCCACCCCAACCAAAAATTTAACA
LALBA	AGCCAGGTCCCTCAGTCAA	ATGGGCCAACCAGTAGTCAA
CSN2	GCTCTTGCAAGGGAGACCAT	TCTGTAATAGATTCCTCACTGCTTG
FTH1	AGAACTACCACCAGGACTCAGA	GTCAAAGTAGTAAGACATGGACAG
SPP1	AACGCCGACCAAGGAAAACT	TGTTGTGGAGGGGTAGGTACA
LYZ	AGGTGTGAGTTGGCCAGAAC	ACACATCCAGTTTGCTAGGC
TMSB10	ACGAGACTGCACGGATTGTTT	TCCGCTTCTCCTGCTCAATG
FABP3	ACTACATGAAGTCACTCGCTCATA	TGAGGGTAGGGGGAAGGTTA
FGFBP1	CAGCCTGGCTCCTGTTGAAT	GGCGTTCACCTTGTTCTGAG
CALML5	TACGAGGAGTTCGCGAGGAT	AGAGTCCCAGCACAAAAGCA
FDCSP	TTTCGCCCACTTCCACCAAT	AGGTGACCAGGTTTATCGTGAC
SOD2	AAACCTCAGCCCTAACGGTG	CACGTTTGATGGCTTCCAGC
TGFBR3	CACTAAGCCCCTTGCTGTGA	CCAGACCATGGAAAATTGGTGG
CSN1S1	CTCACCTGTCTTGTGGCTGT	TGTTTCTCTGCCTGTTCATACC
